# Are You Keeping an Eye on Me? The Influence of Competition and Cooperation on Joint Simon Task Performance

**DOI:** 10.3389/fpsyg.2018.01361

**Published:** 2018-08-03

**Authors:** Jonathan Mendl, Kerstin Fröber, Thomas Dolk

**Affiliations:** Department of Experimental Psychology, University of Regensburg, Regensburg, Germany

**Keywords:** joint action, go–nogo Simon task, reward, cooperation and competition, sequential processing adjustments, referential coding

## Abstract

Social interaction plays an important role in human life. While there are instances that require cooperation, there are others that force people to compete rather than to cooperate, in order to achieve certain goals. A key question is how the deployment of attention differs between cooperative and competitive situation; however, empirical investigations have yielded inconsistent results. By manipulating the (in-)dependence of individuals via performance-contingent incentives, in a visual go–nogo Simon task the current study aimed at improving our understanding of complementary task performance in a joint action context. In the independent condition each participant received what s/he achieves; in the cooperative condition each participant received the half of what both achieved, and in the competitive condition participants were instructed that the winner takes it all. Extending previous findings, we found sequential processing adjustments of the Simon effect as a function of the interdependency (i.e., competition, cooperation) and transition between (i.e., go–nogo requirements) interacting individuals. While sequential processing adjustments of the Simon effect in both the competition and cooperation condition were unaffected when alternating between responsible actors (i.e., nogo–go transition), sequential processing adjustments were enlarged under competition for repeating responsibilities of one and the same actor (i.e., go–go transitions). In other words, the prospect of performance-contingent reward in a competitive context exclusively impacts flexible behavioral adjustments of one’s own actions. Rather than fostering the consideration and differentiation of the other actor, pushing one’s own performance to the limit appears to be the suitable strategy in competitive instances of complementary tasks. Therefore, people keep their eyes on themselves when aiming at beating a co-actor and emerging as the winner.

## Introduction

For humans, it is nearly impossible not to interact with others (Watzlawick et al., 1967). Beyond the significant role of exchanging information and communicating with each other, there are many instances in everyday life that require cooperation (e.g., carrying a table together), while there are others that force people to compete in order to achieve a certain goal (e.g., career position, success in nearly any kind of sport). Although any of those instances are quite familiar to all of us, the question arises: When and to what extent (if anything) do we consider the other person’s actions during social interaction?

Experimental approaches aiming at investigating the underlying mechanisms of social interactions in the laboratory typically use the Simon task (Craft and Simon, 1970). In the standard, two-choice version of the Simon task, participants are asked to respond with a left or right keypress to a particular feature of the stimulus (e.g., the color blue or green), which randomly appears to the left or right side of the screen. If the spatial location of the stimulus corresponds with the spatial location of the assigned response (i.e., compatible trial), responses are typically faster and less error prone. In contrast, if the spatial location of the stimulus and the assigned response differ (i.e., incompatible trial), response times (RTs) and error-rates (ERs) increase. The difference between RTs or ERs in incompatible and compatible trials is called the Simon effect (cf. Simon and Rudell, 1967; for an overview, see Simon, 1990; Lu and Proctor, 1995). This Stimulus–Response (S–R) compatibility effect is typically explained by the dimensional overlap model (Kornblum et al., 1990), which concerns the effect of a match between the task-irrelevant feature of the stimulus (i.e., location) and the required response (i.e., left/right location). Accordingly, the location of the stimulus is assumed to automatically activate a spatially corresponding response, which facilitates task performance on compatible trials and impairs performance on incompatible trials, because resolving the conflict between automatically activated and required responses takes time.

To investigate social interactions, Sebanz et al. (2003) distributed the Simon task among two individuals. Accordingly, each participant was responsible for one stimulus color by operating his/her assigned response-key, while sitting on one side of the screen, converting the two-choice Simon task into a joint go–nogo task. In order to investigate the impact of performing the go–nogo task together with another person, Sebanz et al. (2003) had participants carry out the same go–nogo Simon task individually, thus in the absence of the partner (i.e., individual go–nogo task). While there was no significant S–R compatibility effect in the individual go–nogo condition, there was a compatibility effect when two participants performed the same go–nogo Simon task together, and this finding became known as the social or joint go–nogo Simon effect (JSE; Sebanz et al., 2003; for a review, see Dolk et al., 2014). Interestingly, even though the other person’s (or one’s own nogo) action typically has no direct consequence for the continuation of the experiment (as it simply offers no additional information to facilitate one’s own performance), the mere perception (or expectation) of an alternative action in the joint but not in the individual go–nogo condition seems to impact one’s own task performance. In other words, while it would be an appropriate strategy to concentrate on one’s own task exclusively and completely ignore everything else, people seem unable to do so as soon as there are other (attention attracting) action events in the environment. Thus, the (social) task context seems to modulate the allocation of attention toward the specific S–R associations (cf. Baess and Prinz, 2015). Accordingly, signifying the spatial S–R assignments reintroduces a dimensional overlap of the corresponding dimensions, thereby facilitating (S–R match) or impairing (S–R mismatch) task performance in the joint condition, whereas the lack of an alternative action to one’s own in the individual condition eliminates the need for spatial response coding and thus, there is no dimensional overlap of spatial S–R features.

This explanation nicely fits with the existing theoretical frameworks aiming to explain the emergence of JSEs: The action co-representation (Sebanz et al., 2003) and the referential coding account (Dolk et al., 2013; for spatially inspired accounts, see Guagnano et al., 2010; Dittrich et al., 2012). Grounded in the deeply social nature of human beings, the action co-representation account assumes that one’s own actions and others’ actions are (automatically) represented in a functionally equivalent way. Accordingly, spatially assigned stimuli and responses of the whole task set are considered to be represented, which facilitates task performance in cases of an S–R match, but interferes with performance when there is an S–R mismatch. Based on the Theory of Event Coding (TEC; Hommel et al., 2001), the referential coding account in contrast holds that actions are cognitively represented by codes of their perceivable effects. Given that self- and other-generated actions are represented by the same kind of effect codes, the representation referring to one’s own action needs to be discriminated from all concurrently activated representations in order for the individual to behave appropriately in a given context. Emphasizing the spatial nature of one’s own action as left/right in reference to the other person’s action provides not only a powerful strategy to differentiate alternative action events, it also reintroduces the dimension overlap of spatially defined S–R features. Consequentially, discriminating alternative action events should be more challenging the more similar those events are, resulting in varying effect sizes with varying degrees of similarity (i.e., more similar = larger JSE). These assumptions nicely converge with previous findings showing increased (non-social and social) JSEs with increasing similarity of alternative action events: e.g., Human*_Romantic-Partner_* > Human*_Friend_* (Quintard et al., 2018), Human*_Ingroup_* > Human*_Outgroup_* (Iani et al., 2011; Müller et al., 2011), Human > Puppet (Tsai and Brass, 2007), Human > Computer (Tsai et al., 2008), Robot*_Human-like_* > Robot*_Machine-like_* (Stenzel et al., 2012); Japanese waving cat > Clock > Metronome (Dolk et al., 2013).

Thus, while social variables appear to play an influential role for the co-representation account, the referential coding account highlights the role of the similarity between alternative action events irrespective of its (non-)social nature. In both cases, however, attention allocation toward the spatially distinct alternative actions seem to impact the cognitive representation thereof and, what is more, subsequent behavior (for an attentional focusing account of joint compatibility effects, see Dittrich et al., 2017). This brings the introductory question back into play: When and to what extent do individuals take other people’s (i.e., alternative) actions into account or in other words, which situations require the discrimination of self- and other-generated events? One straightforward approach to tackling this issue is to manipulate the relationship between, or the interdependence of, interacting individuals. While the offending behavior of an intimidating confederate reduced the JSE as compared to a friendly co-actor (Hommel et al., 2009), other studies manipulated the interdependence or in-/dependence of interacting individuals by inducing a more cooperative or competitive relationship via incentives (Ruys and Aarts, 2010; Iani et al., 2011; Ruissen and de Bruijn, 2016).

Using an auditory joint go–nogo Simon task (i.e., reacting to the pitch of a sound), Ruys and Aarts (2010) investigated three reward manipulations to induce different relationships between participants. In advance of the Simon task, participants were instructed that either: (i) the ten best performing subjects will win ten euros (independent group), (ii) each actor of the five best performing teams will earn five euros (cooperative group), or (iii) ten team winners will be randomly selected for the ten euros reward (competition group). Results revealed a larger JSE in the dependent groups (i.e., the cooperative and competitive groups), in comparison to the independent group. This finding has been taken to suggest that interdependency leads to a stronger attentional focus on the partner, and therefore to stronger shared representations and to a larger JSE. Iani et al. (2011) improved the definition of competition by instructing participants that each actor of the best performing team will be rewarded with five euros in the cooperative group, or that only the team winner will receive five euros as a reward in the competitive group. In sharp contrast to Ruys and Aarts (2010), the results revealed a significant difference between dependent groups, with a JSE in the cooperative group but no JSE in the competitive group. Thus, considering the co-actor might selectively occur when aiming to beat others as a team, but the exact opposite takes place when having an opponent. Further support for the crucial role of the exact type of interdependency is provided by a study of Ruissen and de Bruijn (2016) showing a smaller JSE in a group of participants that played Tetris in a competitive as compared to a cooperative (or isolated, i.e., solo) style of social interaction prior to the joint go–nogo Simon task performance.

Even though previous findings suggest more attention to the other’s actions in a cooperative compared to a competitive relationship, there are several methodological issues that warrant further investigation in order to fully understand the processes that drive these socially driven flexible adjustments of attention allocation. In addition to investigating go–nogo Simon task performance in an independent, cooperative and competitive joint condition, the present study made use of an individual go–nogo task at the beginning of the experiment to provide a valuable reference for the resulting JSEs. Furthermore, the definitions of the terms cooperation and competition were further adjusted from those used by Iani et al. (2011). Here, cooperation instructions emphasize team work for achieving a common goal (and not a cooperative competition against unspecified others as in Iani et al., 2011), while competition instructions more clearly highlight the battle of opponents (i.e., the winner takes it all) to achieve the individual goal of emerging as the winner. To further amplify the effect of interactive contexts, reward was given for every (correct and fast enough) trial.

More importantly, however, the present study followed the recommendation of Liepelt et al. (2013) in taking sequential processing adjustments (i.e., trial-by-trial dependencies) in go–nogo Simon task performance into account to achieve a more detailed picture of the underlying processes (cf. Liepelt et al., 2013). That is, compatibility effects like Flanker, Simon, and Stroop are typically smaller after incompatible compared to compatible trials (Gratton et al., 1992; for a review, see Egner, 2014). This conflict adaptation or Gratton effect is considered to reflect reduced interference as a consequence of cognitive control already being up-regulated in the trial following an incompatible (conflicting) trial (Botvinick et al., 2001; for a review, see Botvinick, 2007). Liepelt et al. (2011) emphasized this effect in an individual and joint go–nogo Simon task, while highlighting the role of sequential processing adjustments for different types of trial-to-trial transitions. These can either be (i) nogo–go transitions, where the participant had to withhold a response in the previous trial but is required to respond in the current trial, or (ii) a go–go transition, in which the participant was required to respond in both the previous and the current trial. Interestingly, while sequential adaptation effects were stronger for nogo–go transitions than for go–go transitions in both tasks, these where overall smaller in the individual go–nogo task suggesting additional between-person discrimination (i.e., whose turn is it?) processes in the course of a nogo–go transition (Liepelt et al., 2011; Yamaguchi et al., 2016). For the present study, those transition effects are particularly interesting as they can indicate changes of the attentional focus, by signifying differences in sequential processing adjustments after one’s own compared to the partner’s response. Considering a positive to neutral and thus, rather cooperative style when engaged in social interactions with others as default (Iani et al., 2011), constantly attending to the partner’s action enables flexible adjustments to the other in order to achieve the common goal together. The critical question, however, is whether and (if any) to which extent participants’ attention is drawn to one’s own or the other’s performance in competitive interactive contexts. In other words, do participants in a competitive relationship apply a self- or other-referenced focus (Poortvliet and Darnon, 2010). While the latter is suggested to be applied when aiming to outperform the other, the former might be more suitable in particular (task) circumstances in which constant monitoring and comparing one’s own and the others performance is quite demanding, thus resource-consuming. That is, attending to the co-actors’ action might simply not be an appropriate strategy for participants who are trying to improve their own performance, because they have little to no direct influence on changing their opponent’s actions in a go–nogo Simon task. The only thing they can influence in such a situation is their own action, which should result in a self-referenced focus (Iani et al., 2011), leading to no reliable nogo–go, but notable go–go transition effects, because they refer to sequential processing adjustments after one’s own response.

Based on this framework, the present study investigated the processes underlying flexible adjustments to the contextual challenge of either cooperating or competing for reward when interacting with others. To that end, participants performed an individual go–nogo Simon task at the beginning of the experiment followed by a go–nogo joint Simon task with the prospect of reward that was largely independent of the co-actor’s performance. That is, prior to the joint go–nogo Simon task, participants were instructed that each participant in the pair would receive the amount of reward that s/he actually earned for fast and correct responses on their own (i.e., maximizing my own reward irrespective of the co-actor; *independent goal*). Prior to the final part of the experiment participants were informed that the amount of reward will be equally divided between both participants (i.e., maximizing the total reward sum together with the co-actor; *shared goal = cooperation*) or that the participant that earned the most reward will receive the whole amount of reward, including the amount earned by the other person (i.e., being better than the co-actor to win and not to lose in the end the whole reward sum; *competitive goal*; see **Table 1** for an overview of the experimental procedure). If participants in the competition group develop an other-referenced focus, we would expect larger JSEs and sequential processing adjustments after nogo–go transitions compared to the cooperative group. On the other side, if participants in the competitive group apply a self-referenced focus, they should show a smaller JSE, and larger adjustments after go–go transitions compared to the cooperative group. Results in the individual go–nogo Simon task and the independent joint go–nogo Simon task should be in line with previous findings showing no go–nogo Simon effect but significant sequential processing adjustment in the former and significant effects in both measures in the latter condition.

**Table 1:** Procedure of an experimental session.

	Session and experimental setup^a^	Reward manipulation
**Task 1**	*Individual go*–*nogo Simon task*	*Individual baseline*
	- Participants are seated on separate computers (**Figure 1A**)	- No reward manipulation
	- Each participant responds to only one assigned stimulus	- Determination of individual RT thresholds for performance-contingent reward in the following tasks

**Task 2**	*Independent joint go*–*nogo Simon task*	*Independent performance-contingent reward*
	- Both participants are seated in front of the same computer (**Figure 1B**)	- Each correct and fast enough trial is rewarded with 4 cents
	- Each participant responds to only one assigned stimulus	- An incorrect response is punished with a loss of 2 cents that is credited to the other participant
		- Each participant gets the amount of money s/he earned on her/his own (independent goal)

**Task 3**	*Dependent joint go*–*nogo Simon task*	**Competition:**
		*Dependent performance-contingent reward*
	- Both participants are seated in front of the same computer (**Figure 1B**)	- Each correct and fast enough trial is rewarded with 4 cents
	- Each participant responds to only one assigned stimulus	- An incorrect response is punished with a loss of 2 cents that is credited to the other participant
		- The participant who earned the most receives the total sum of reward earned by both participants (shared goal)
		**Cooperation:**
		*Dependent performance-contingent reward*
		- Each correct and fast enough trial is rewarded with 4 cents
		- An incorrect response is punished with a loss of 2 cents - The total amount of reward is equally divided between both participants (competitive goal)


*^*a*^Tasks 1 and 2 comprised one training block of 16 trials and two testing blocks of 128 trials each, while Task 3 contained only two testing blocks of 128 trials each.*

## Materials and Methods

### Participants

Forty-eight right-handed undergraduate students of the University of Regensburg (44 female; *M*_age_ = 19.7, *SD*_age_ = 1.9, *R*_age_ = 18–28 years) participated in the present study.^1^^,^^2^ Participants had normal or corrected-to-normal vision and were naive with regard to the hypothesis of the experiment. Participants gave their written informed consent before their inclusion in the study in accordance with the ethical standards of the German Psychological Society (DGPs; 2016) and the 1964 Declaration of Helsinki. According to the DGP’s ethics commission, an institutional research board’s ethical approval is only required if (i) research carries additional risk beyond daily activities or (ii) any funding is subject to such an ethical review. No such requirements were present for this study. After the session, all participants were debriefed and rewarded with partial course credit. Participants were tested in pairs and did not know each other prior to the experiment. Data from three participants were excluded due to mean reaction times or error rates of more than 2.5 SDs from the task mean.

### Material and Procedure

For the present go–nogo Simon tasks, a green and a blue circle with a diameter of one centimeter were used as stimuli (0.96° × 0.96°; cf. Hommel et al., 2009). They were presented 8.75 cm to the left or the right of the center (eccentricity of 8.7° visual angle) using E-Prime 2.0 (Psychology Software Tools, Sharpsburg, PA, United States).

**FIGURE 1:**
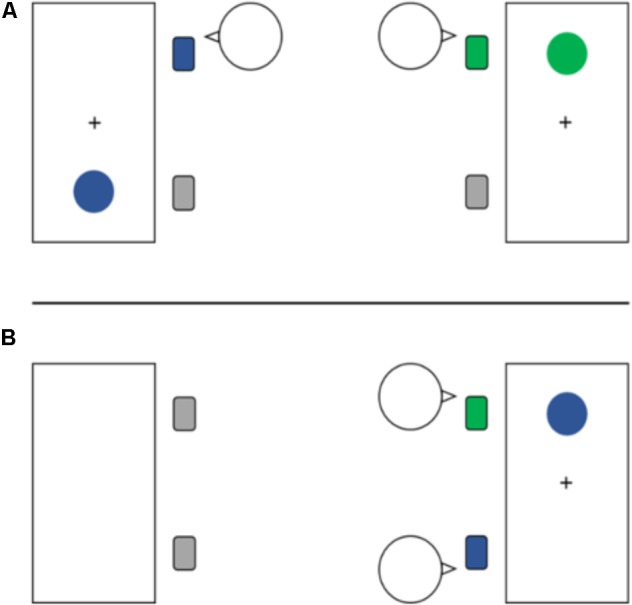
Experimental setup in the individual go–nogo Simon task (Task 1; **A**) and in the joint go–nogo Simon tasks (Tasks 2 and 3; **B**). In both go–nogo Simon task contexts **(A,B)** the participants are required to respond to their assigned stimulus (blue circle, person on the right, incompatible trial; green circle, person on the left, compatible trial) by operating the response key in front of them. Stimulus–Response assignments as well as spatial position of the participants were counterbalanced across participants but held constant across the tasks. Whereas in the individual go–nogo Simon task (1; **A**) participants worked on adjacent computers, in the joint go–nogo Simon tasks (2 and 3; **B**) both participants sat in front of one computer.

Upon arrival at the laboratory, pairs of participants were informed about the three consecutive segments of the experiment, namely performing the first task alone and the following two together with the other person. Prior to the instruction phase of the first (an individual go–nogo Simon) task, both participants were seated at their respective workspaces composed of two seats in front of a computer with a 17-inch monitor (display resolution at 1,024 × 768 pixels) at a viewing distance of approximately 60 cm (**Figure 1A**). To enable a consistent spatial arrangement of left/right chair and corresponding response across all tasks, participants in Task 1 sat back-to-back leaving the second chair at each workspace empty. That is, while the participant assigned to the left workspace was seated in the left chair and responded via the left response key (i.e., the “Y”-key on a QWERTZ-keyboard), the participant assigned to the right workspace was seated in the right chair and operated the right response (“M”-) key (**Figure 1A**). Both participants were instructed to put their right index finger on the respective response key while leaving their left hand underneath the table on their left thigh.

To familiarize participants with the task, the experiment started with an instruction phase (∼5 min) including the presentation of the two stimuli, their assignment to each participant and a training of 16 trials in total. After the instruction phase was completed, the experimental phase of Task 1 started. There were two blocks of 128 trials, which equally often contained each stimulus (blue vs. green) with each S–R mapping (compatible vs. incompatible). This task was used to calculate the individual reaction time (RT) threshold for performance-contingent reward receipt in Task 2 and Task 3. The threshold was determined by the 0.33-quantile of all correct responses sorted from fast to slow (cf., Fröber and Dreisbach, 2014, 2016). To maintain vigilance throughout the whole experiment, short self-paced breaks between blocks and a 2-min break between Task 1 and Task 2 outside the laboratory were provided.

Following Task 1 and a recovering break, participants reentered the lab to continue with the second segment, a joint go–nogo Simon task with performance-contingent reward. In order to keep S–R assignments and responsibilities consistent with Task 1, both participants were asked to take their respective seat of either the left or the right workspace (counterbalanced across pairs of participants). Thus, while the workspace remained the same for one participant, the other had to change, but the spatial assignment of chair and response-key remained the same (see **Figure 1B**). After participants were reminded about stimuli and respective assignments, they were instructed about the possibility of earning four cents for every correct and very fast response (i.e., faster than the individual RT threshold) and irrespective of the partner’s performance, to explicitly emphasize an independent relationship between interacting individuals. Note, however, to keep the task fair, a participant would lose two cents in case of an error and the partner would gain these two cents, because an error of one participant always represented a lost opportunity for the other participant to gain reward. After the instruction, participants performed 16 more training trials in order to get familiar with the task and to give the participants a feeling of about how fast they have to react to receive the reward. Following this short training, participants got feedback about the amount of money they would have received before the experimental phase of Task 2 started. As in Task 1, participants had to perform 256 testing trials divided in two blocks and they received feedback about the earned amount of money after each block.

After Task 2, participants continued with the third segment, again a joint go–nogo Simon task with performance-contingent reward. The procedure was similar to the last task with the following exception: In contrast to Task 2, the amount of reward each participant received at the end of Task 3 depended upon the interactive mode, that is, whether participants competed or cooperated. More precisely, in the cooperative group, participants were instructed that the amount of reward both participants earned during the course of the experiment will be equally divided at the end of the experiment, thereby aiming to emphasize to work as a team for a common goal. Consequentially, error punishment was changed such that wrong responses still led to a loss of two cents, but the amount was not added to the partner’s score. In the competitive group, however, participants were informed about “the winner takes it all principle,” aiming to increase the challenge of receiving the desired goal. Thus, the participant who earned the most during the course of the experiment will receive not only her/his own reward but also the amount of the reward earned by the co-actor. Accordingly, error punishment was the same as in Task 2: Producing an error resulted in a loss of two cents and a gain of two cents for the opponent. After this instruction, the experimental phase started immediately (i.e., without further training) with 256 testing trials, divided into two blocks and a feedback about the earned amount of money after each block (see **Table 1** for an overview of the experimental procedure).

Each trial of the different Simon tasks started with a fixation cross in the center of the screen for 250 ms followed by the imperative stimulus (i.e., a blue or a green circle) presented to either the left or the right side of the screen for 1,000 ms or until a response was given. If the response was correct and fast enough, the next trial started after an inter-trial interval (ITI) varying randomly between 500 ms and 1,200 ms in steps of 100 ms. If not, the German words for error (i.e., “Falsch!”) or too slow (i.e., “Zu langsam!”) were displayed on the screen for 1,000 ms, thus extending the ITI for about 1,500–2,200 ms in Task 1 and the training trials of Task 2. There was no error feedback in the testing trials of Task 2 or in Task 3.

After the three sessions of go–nogo Simon task performances, participants were asked to complete three computerized questionnaires at their own workspace, respectively. The first questionnaire involved the “Inclusion of Other in the Self” (IOS) scale (Aron et al., 1992), a single-item pictorial measure for perceived interpersonal connectedness. Here, participants are asked to indicate which of the seven pictures best describes their own relationship with the co-actor. The IOS was aimed to provide a proof of concept for the interactive mode (i.e., competitive vs. cooperative) in Task 3. Following the IOS, participants answered six questions about their focus of attention (I fully concentrated on my own task in the last two blocks; In the last two blocks of the experiment, I kept a close eye on the other participant’s reaction; A sort of rhythm developed between my reaction and the reaction of the other participant; I tried to ignore the reaction of the other participant; The reaction of the other participant strongly distracted me from my task; I strongly concentrated on the other participant’s task). Participants could answer on a five-point scale with possible answers “very true for me,” “somewhat true for me,” “neutral,” “somewhat false for me,” and “very false for me.” Those questions were intended to measure the attentional focus of the subjects in Task 3. The last questionnaire was the BIS/BAS Scale (Carver and White, 1994), which has 24 items in form of statements indicating approach and avoidance motivation. Participants responded on a four-point scale with “very true for me,” “somewhat true for me,” “somewhat false for me,” or “very false for me.” The reward responsiveness subscale of the behavioral approach system in particular could influence participants in the rewarded Tasks 2 and 3.

### Design

A 2 (Compatibility*_N_*: compatible, incompatible) × 2 (Compatibility_*N*-1_: compatible, incompatible) × 2 (Transition: go–go, nogo–go) × 2 (Block: 1, 2) × 2 (Group: cooperation, competition) mixed analysis of variance (ANOVA) was conducted for each of the three tasks. The within-subjects factors were compatibility in the current trial (Compatibility*_N_*), compatibility in the previous trial (Compatibility_*N*-1_), Transition and Block, while Group was a between-subjects factor. In order to investigate the impact of the specific interdependence (i.e., the in-/dependence) on interacting individuals in the go–nogo Simon task, we included the within-subjects factor Task (2, 3) in the original 2 × 2 × 2 × 2 × 2 ANOVA (Supplementary Table S1).

## Results

### Data Preprocessing

For statistical analysis, we excluded the first trial of each block, erroneous and post-error trials (together 3.2%) as well as trials with RTs lower than 100 ms and RTs that were more than 3 SDs from the individual cell mean (together 0.4%). Error rates were rather low 1.3%, and were not analyzed further. The significance criterion was set to *p* < 0.05.

### RT Analysis for Task 1 (Individual Go–Nogo)

**FIGURE 2:**
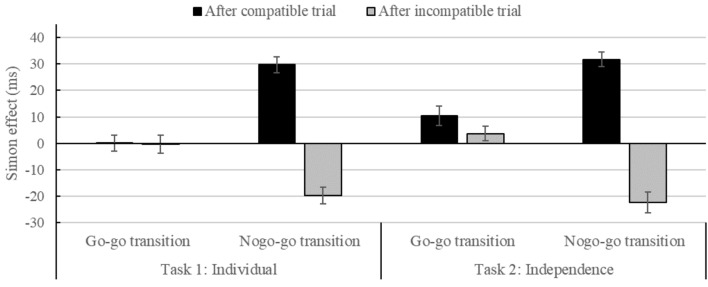
Simon effect (RT_incompatible_ – RT_compatible_) in ms as a function of Compatibility_N-1_ (compatible, incompatible) and Transition (go–go, nogo–go) in the individual go–nogo Simon task (1) and in the joint go–nogo Simon task (2) with independent reward. Error bars represent one standard error of the means.

The 2 × 2 × 2 × 2 × 2 ANOVA revealed no significant main effect of Compatibility*_N_*, *F*(1,43) = 1.42, *p* > 0.05. However, there was a sequential adaptation effect as indicated by a significant interaction of Compatibility*_N_* and Compatibility_*N*-1_, *F*(1,43) = 93.61, *p* < 0.001, $\eta_{p}^{2}$ = 0.69. The Simon effect was smaller after incompatible than after compatible trials [-10 vs. 15 ms; *t*(44) = 9.57, *p* < 0.001, *d* = 1.54; for descriptive details, see **Table 2**]. This interaction was further qualified by a higher order interaction between Transition, Compatibility*_N_* and Compatibility_*N*-1_, *F*(1,43) = 61.55, *p* < 0.001, $\eta_{p}^{2}$ = 0.59. As can be seen in **Figure 2**, this interaction can be explained by larger sequential processing adjustments of the Simon effect for nogo–go transitions than for go–go transitions [49 vs. 0 ms; *t*(44) = 7.76, *p* < 0.001, *d* = 1.79]. The significant main effect of Block, *F*(1,43) = 9.54, *p* < 0.01, $\eta_{p}^{2}$ = 0.18, indicated that participants responded faster in the first (*M* = 329, *SD* = 32) than in the second block (*M* = 337, *SD* = 32). The significant main effect of Transition, *F*(1,43) = 16.10, *p* < 0.001, $\eta_{p}^{2}$ = 0.27, showed faster RTs for go–go (*M* = 328, *SD* = 34) than for nogo–go transitions (*M* = 338, *SD* = 30). This was further qualified by a significant interaction between Block and Transition, *F*(1,43) = 25.49, *p* < 0.001, $\eta_{p}^{2}$ = 0.37, revealing a larger Transition effect in the second than in the first block [16 vs. 4 ms; *t*(44) = 5.09, *p* < 0.001, *d* = 0.68]. The significant two-way interaction between Block and Compatibility_*N*-1_, *F*(1,43) = 6.94, *p* < 0.05, $\eta_{p}^{2}$ = 0.14, indicated faster RTs after incompatible trials compared to compatible trials in block 2 than in block 1 [4 vs. -1 ms; *t*(44) = 1.16, *p* < 0.05, *d* = 0.51]. The interaction between Transition and Compatibility*_N_*, *F*(1,43) = 4.56, *p* < 0.05, $\eta_{p}^{2}$ = 0.10, showed a larger Simon effect for nogo–go as compared to go–go transitions [5 vs. 0 ms; *t*(44) = 2.17, *p* < 0.05, *d* = 0.33]. All other main effects or interactions did not reach significance (all *F*s < 3.11, all *p*s > 0.084).

**Table 2:** Response times (SD) in milliseconds for compatible and incompatible trials as a function of task (Individual go–nogo, Independent go–nogo, Dependent go–nogo) and transition.

	Compatible	Incompatible
Individual go–nogo Simon task (Task 1) Go–go transition		
After compatible trial	329 (36)	329 (36)
After incompatible trial	328 (36)	328 (36)
Nogo–go transition		
After compatible trial	325 (30)	354 (35)
After incompatible trial	347 (29)	327 (33)
Independent joint go–nogo Simon task (Task 2) Go–go transition		
After compatible trial	296 (29)	306 (30)
After incompatible trial	296 (28)	300 (29)
Nogo–go transition		
After compatible trial	292 (27)	323 (25)
After incompatible trial	322 (29)	300 (29)
Dependent joint go–nogo Simon task (Task 3) Cooperative group		
Go–go transition		
After compatible trial	296 (27)	305 (29)
After incompatible trial	295 (28)	299 (26)
Nogo–go transition		
After compatible trial	295 (27)	334 (28)
After incompatible trial	326 (26)	303 (26)
Competitive group Go–go transition		
After compatible trial	287 (27)	299 (29)
After incompatible trial	292 (28)	285 (26)
Nogo–go transition		
After compatible trial	287 (27)	319 (28)
After incompatible trial	314 (26)	293 (26)


### RT Analysis for Task 2 (Independent Joint Go–Nogo)

The respective 2 × 2 × 2 × 2 × 2 ANOVA revealed a significant main effect of Compatibility*_N_*, *F*(1,43) = 7.85, *p* < 0.01, $\eta_{p}^{2}$ = 0.15, indicating faster responses for compatible compared to incompatible trials (*M_compatible_* = 301, *SD_compatible_* = 27, *M_incompatible_* = 307, *SD_incompatible_* = 26). As in Task 1, there was a sequential adaptation effect as indicated by a significant interaction of Compatibility*_N_* and Compatibility_*N*-1_, *F*(1,43) = 172.07, *p* < 0.001, $\eta_{p}^{2}$ = 0.80, with a smaller Simon effect after incompatible than after compatible trials [-9 vs. 21 ms; *t*(44) = 12.96, *p* < 0.001, *d* = 1.90; **Table 2**], as well as between Compatibility*_N_*, Compatibility_*N*-1_ and Transition, *F*(1,43) = 41.94, *p* < 0.001, $\eta_{p}^{2}$ = 0.49 (for a comparison between Tasks, Supplementary Table S1). As can be seen in **Figure 2**, the sequential processing adjustments were larger for nogo–go compared to go–go transitions [54 vs. 7 ms; *t*(44) = 6.55, *p* < 0.001, *d* = 1.64]. The significant main effect of Transition, *F*(1,43) = 34.28, *p* < 0.001, $\eta_{p}^{2}$ = 0.44, showing faster RTs for go–go (*M* = 300, *SD* = 27) than for nogo–go transitions (*M* = 309, *SD* = 24), varied as a function of Block, *F*(1,43) = 13.37, *p* = 0.001, $\eta_{p}^{2}$ = 0.24, such that there was a smaller Transition effect in block 1 as compared to block 2 [5 vs. 14 ms; *t*(44) = 3.69, *p* < 0.01, *d* = 0.65]. The interaction between Transition and Compatibility_*N*-1_, *F*(1,43) = 6.37, *p* < 0.05, $\eta_{p}^{2}$ = 0.13, indicated faster RTs after compatible trials compared to incompatible trials for nogo–go transitions than for go–go transitions [4 vs. -3 ms; *t*(44) = 2.55, *p* < 0.05, *d* = 0.59]. Furthermore, the interaction between Block, Compatibility_*N*-1_ and Group, *F*(1,43) = 4.09, *p* < 0.05, $\eta_{p}^{2}$ = 0.09, was significant, indicating larger RT-differences after compatible trials compared to incompatible trials between blocks in both the competitive and the cooperative group [6 ms vs. 3 ms; *t*(43) = 2.02, *p* < 0.05, *d* = 0.60]. No other main effects or interactions reached significance (all *F*s < 2.24, all *p*s > 0.142).

### RT Analysis for Task 3 (Dependent Joint Go–Nogo)

**FIGURE 3:**
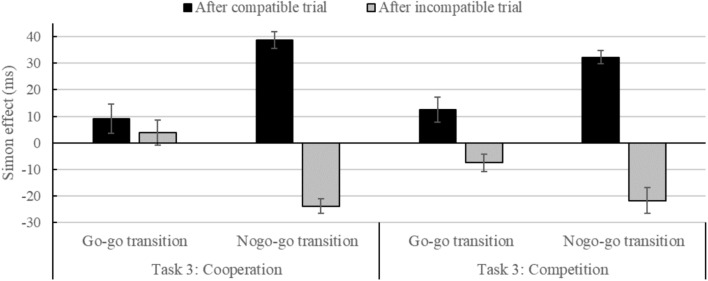
Simon effect (RT_incompatible_ – RT_compatible_) in ms as a function of Compatibility_N-1_ (compatible, incompatible), Transition (go–go, nogo–go), and Group (Cooperation, Competition) in the joint go–nogo Simon task (3) with dependent reward (Cooperation: Equally divided, Competition: “the winner takes it all”). Error bars represent one standard error of the means.

In the RT analysis of task 3, a significant main effect of Compatibility*_N_* was observed, *F*(1,43) = 8.40, *p* < 0.01, $\eta_{p}^{2}$ = 0.16, indicating faster responses for compatible than for incompatible trials (*M_compatible_* = 299, *SD_compatible_* = 25, *M_incompatible_* = 305, *SD_incompatible_* = 25; **Table 2**). The interaction between Compatibility*_N_* and Compatibility_*N*-1_, *F*(1,43) = 145.02, *p* < 0.001, $\eta_{p}^{2}$ = 0.77, with a smaller Simon effect after incompatible than after compatible trials [-12 vs. 23 ms; *t*(44) = 12.14, *p* < 0.001, *d* = 2.22], and the interaction between Compatibility*_N_*, Compatibility_*N*-1_ and Transition, *F*(1,43) = 71.64, *p* < 0.001, $\eta_{p}^{2}$ = 0.63, with larger sequential processing adjustments for nogo–go compared to go–go transitions [58 vs. 12 ms; *t*(44) = 8.19, *p* < 0.001, *d* = 1.69], were further qualified by a higher order interaction between Compatibility*_N_*, Compatibility_*N*-1_, Transition, and Group, *F*(1,43) = 4.60, *p* < 0.05, $\eta_{p}^{2}$ = 0.10. As can be seen in **Figure 3**, this four-way interaction can be explained by a significant sequential adaptation of the Simon effect for go–go transitions in the competition group [20 ms; *F*(21) = 11.38, *p* < 0.01, $\eta_{p}^{2}$ = 0.35], but non-significant sequential adaptation of the Simon effect for go–go transitions in the cooperative group [5 ms; *F*(22) = 0.63, *p* = 0.438]. There was a smaller Simon effect after incompatible trials than after compatible trials for go–go transitions in the competitive group [-7 vs. 13 ms; *t*(21) = 3.37, *p* < 0.01, *d* = 1.04], but not in the cooperative group [4 vs. 9 ms; *t*(22) = 0.79, *p* = 0.438], and a smaller Simon effect after incompatible trials than after compatible trials for nogo–go transitions in the competitive group [-22 vs. 32 ms; *t*(21) = 10.11, *p* < 0.001, *d* = 2.99] and the cooperative group [-24 vs. 39 ms; *t*(22) = 14.37, *p* < 0.001, *d* = 4.17]. However, the interaction between Compatibility and Group did not reach significance [*F*(1,43) = 0.66, *p* > 0.05]. Furthermore, the main effect of the Transition reached significance, *F*(1,43) = 44.41, *p* < 0.001, $\eta_{p}^{2}$ = 0.51, suggesting faster RTs for go–go (*M* = 295, *SD* = 24) than for nogo–go transitions (*M* = 309, *SD* = 25). The interaction between Transition and Compatibility_*N*-1_, *F*(1,43) = 4.11, *p* < 0.05, $\eta_{p}^{2}$ = 0.09, indicated faster RTs after incompatible trials compared to compatible trials for go–go transitions than for nogo–go transitions [4 vs. 0 ms; *t*(44) = 2.05, *p* < 0.05, *d* = 0.41]. All other main effects or interactions were not significant (all *F*s < 3.66, all *p*s > 0.062).

### Between-Task Analysis and Questionnaires

**FIGURE 4:**
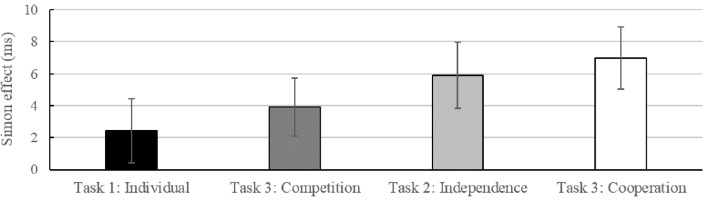
Simon effect (RT_incompatible_ – RT_compatible_) in ms for all conditions (Task 1: Individual, Task 3: Competition, Task 2: Independence, Task 3: Cooperation). Error bars represent one standard error of the means.

Although the go–nogo Simon effect increased as a function of the interdependence of interacting individuals – competition < independence < cooperation (**Figure 4**) – the respective interaction between the factors Compatibility*_N_*, Group and Task did not reach significance, *F*(1,43) = 1.82, *p* = 0.184 (Supplementary Table S2). The rest of this analysis’ results brought no further information to the findings detailed above. In the analyses of the questionnaires, *T*-tests showed no significant difference between groups on the IOS scale, the mean response to the strategy questions, or the BAS reward responsiveness score (all *t*s < 0.52, all *p*s > 0.604). Furthermore, there were no significant Spearman correlations between Group, IOS response, mean Strategy response and BAS reward responsiveness (all *p*s > 0.689).

## Discussion

The present research investigated the influence of in-/dependence on interacting individuals in joint go–nogo Simon tasks. More precisely, reward prospect for each (fast and correct) trial for each participant was context-dependently manipulated to enable the instantiation of different interdependencies between co-acting participants. That is, participants were, prior to the joint go–nogo Simon task, instructed that (i) each participant of the pair would receive the amount of reward that s/he actually earned for fast and correct responses on their own (i.e., independent reward) or (ii) that the amount of reward would be equally divided between both participants (cooperative dependence) or (iii) that the participant that earned the most reward would receive the whole amount of reward, including the amount earned by the other person (competitive dependence). Extending previous findings, the present study revealed sequential processing adjustments of the go–nogo Simon effect as a function of the interdependency of (i.e., competition, cooperation) and transition between interacting individuals (i.e., go–nogo requirements). While sequential processing adjustments of the Simon effect in both the competition and cooperation condition were unaffected when alternating between responsible actors (i.e., nogo–go transition), sequential processing adjustments were enlarged under competition for repeating responsibilities of one and the same actor (i.e., go–go transitions). In other words, the prospect of performance-contingent reward in a competitive context exclusively impacts flexible behavioral adjustments of one’s own actions. Rather than fostering the consideration and differentiation of the other actor (i.e., other-referenced frame), pushing one’s own performance to the limit appears to be the suitable strategy in competitive instances of complementary tasks (i.e., self-referenced frame; Poortvliet and Darnon, 2010). Therefore, people keep their eyes on themselves when aiming at beating a co-actor and emerging as the winner.

Even though the present findings provide further valuable insight into the mechanisms driving flexible adjustments to changing contextual challenges when interacting with others (Liepelt et al., 2011; Yamaguchi et al., 2016), two critical aspects need further elaboration to close the gaps in the literature. One concerns the obviously crucial role of defining in-/dependence, and the other why the present study failed to show a modulation of the joint go–nogo Simon effect as a consequence of the interdependency of interacting individuals beyond the sequential trial-to-trial processing adjustments. First, how in-/dependence is defined appears to be particularly important for how attention is deployed. Ruys and Aarts (2010) found a JSE difference between dependence and independence, but no difference between the dependent conditions (cooperation, competition). In contrast, in our study, the sequential processing adjustments indicate a different attentional focus between cooperation and competition. In this way, the findings are in line with the study of Iani et al. (2011) as well as Ruissen and de Bruijn (2016), which show a distinction between cooperative and competitive dependence on the level of the JSE. An explanation for this inconsistency lies in the rather vague definitions of cooperation and competition in the study of Ruys and Aarts (2010). While they manipulated competition by rewarding 10 randomly selected team winners, Iani et al. (2011) improved this manipulation by rewarding one winner within each team. This distinction could explain the discrepancy of the JSE modulations between the different studies. However, only the present study shaped the cooperative relationship without alluding to unspecified other teams, while in the study of Iani et al. (2011) as well as Ruys and Aarts (2010), reward was given to the best performing team, which induced a competitive relationship with other teams. In this aspect, the present definition covers the complex construct of cooperative interaction in a proper way by solely manipulating the relationship within the team.

**FIGURE 5:**
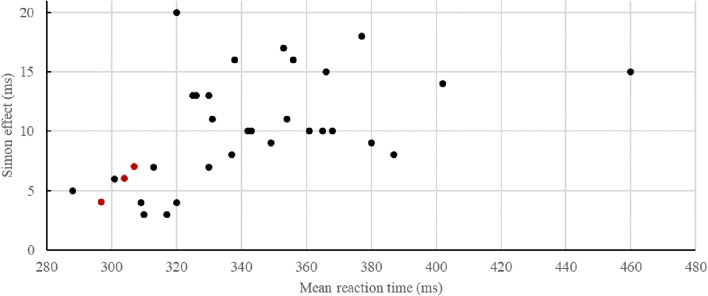
Simon effect (RT_incompatible_ – RT_compatible_) in ms as a function of the mean reaction time in previous studies using visual joint go–nogo Simon tasks (for more details see “Discussion” and Footnote 1). The red data points represent the results of the joint conditions of the present study (from left to right: Task 3: Competition, Task 2: Independence, Task 3: Cooperation).

More interestingly, however, the modulation of the JSE as a consequence of the interdependency manipulation found by Iani et al. (2011) and by Ruissen and de Bruijn (2016) did not reach significance in the present study, even though descriptively the results point in the same direction of a smaller JSE in the competitive as compared to the cooperative group (**Figure 4**). One reasonable explanation concerns the specific reward manipulations in the present study. Highlighting the significance of each trial via reward prospect for each correct and fast enough trial (i.e., each response in the fastest third of all correct RTs in the individual go–nogo Task 1) seems to have pushed task performance to the ceiling, leading overall as well as within each task to decreasing RTs and smaller JSEs, and thus to not much room for significant variability. Interestingly, this observation stands in sharp contrast to what is typically found for the standard (i.e., two choice) Simon task, namely increasing Simon effects with decreasing RTs (Hommel, 1993). Even though Hommel et al. (2009) use this pattern of a standard Simon task to reject the possibility that the non-significant JSE in the negative relationship condition, where participants reacted alongside an intimidating confederate, is solely driven by response speed, the attenuation of the JSE with decreasing RTs is perfectly in line with the present and previous findings. The Google scholar citation index for the initial JSE study of Sebanz et al. (2003) on April 1st 2018 revealed 17 viable studies that used a visual joint go–nogo Simon task with two participants sitting next to and sharing the same workspace with each other (**Figure 5**)^3^. The positive correlation of *r* = 0.52 (*p* < 0.01) indicates that smaller RTs were predictive of smaller JSEs. Thus, in contrast to Hommel et al. (2009) and the findings in a standard Simon task showing increasing Simon effects with decreasing RTs (Hommel, 1993), go–nogo Simon effects are attenuated with increasing RTs, suggesting the involvement of different processes in the emergence of those two effects. In a standard Simon task with two different stimulus features and two response alternatives, the irrelevant spatial feature of the stimulus overlaps with the spatial feature of the response and is considered to automatically activate a representation of the spatially corresponding response. Interestingly, if participants react more slowly, response code activation induced by the location, which may conflict with the correct response, seems to decay over time, leading to smaller Simon effects (Hommel, 1994). If participants try to maximize performance and react as fast as possible, conflict resolution in a two-choice task takes up extra time in incompatible trials, thereby leading to larger Simon effects. In contrast, in a joint go–nogo Simon task, there are substantially different processes at play (Dolk and Prinz, 2016). Participants have only one response key and need to respond to only one of two stimulus features, thus a selective rather than a (two-) choice reaction is required on any given trial. While one’s own alternative response hand in the two-choice Simon task provides a reference for spatial response coding that signifies spatial S–R overlap and thus elaborated Simon effects, this attention allocation toward task inherent S–R assignments seems to require a salient alternative (social or non-social) event in the individual’s workspace (Dolk et al., 2011, 2013, 2014). Accordingly, if the participant tries to react as quickly as possible by applying top-down control to primarily focus on one’s own task, this might be responsible for smaller Simon effects with faster RTs. In any case, it will be important for future work to clarify the different underlying processes that govern the emergence of the standard and the (joint) go–nogo Simon effect when performance is pushed to the limit.

The most noteworthy finding of the present study is that the flexible adjustments of attention allocation differ based on the dependencies of interacting individuals, as shown in the four-way interaction between compatibility in the present trial, compatibility in the last trial, trial-to-trial transition, and group. Sequential trial-by-trial processing adjustments were enlarged under competition for repeating responsibilities of the same actor (go–go transitions), which implies a stronger focus on one’s own task. This self-focus may be an attempt to maximize performance in order to have a higher chance of getting the reward. This finding nicely converges with behavioral and electrophysiological results of de Bruijn et al. (2008) showing that disengaging from the partner can be beneficial for one’s own performance. Together with the present result, these findings provide compelling evidence against the view of Ruys and Aarts (2010) arguing that the type of relationship between the participants is irrelevant for the emergence of shared representations and, as long as there is interdependence, participants attend to the partner’s performance. Compared to the finding of Ruissen and de Bruijn (2016) as well as Iani et al. (2011), who observed differences on the level of the JSE, the present findings provide an even more complex distinction of different types of interdependencies between interacting individuals derived by attention allocation, namely a stronger focus on one’s own performance under competition. This interplay between attention allocation and the size of the JSE is perfectly in line with various experiments. For example, Colzato et al. (2012a) found that participants, whose attention was drawn to interdependence by circling interdependent pronouns (e.g., we, our) in essays, show a larger JSE compared to participants with a self-centered focus after having circled independent pronouns (e.g., I, me). Similarly, Colzato et al. (2013) found a larger JSE in a group of participants after a divergent thinking task, which lead to a broader attentional focus, compared to a convergent thinking task that promoted an exclusive cognitive-control state. All of those findings support the view, that, if the context at hand enables one narrowing the focus to one’s own task, the JSE is typically decreased. As such, the (joint) go–nogo Simon task appears to be a viable tool to investigate flexible adjustments of attention allocation governing self-other integration when interacting with others (cf. Colzato et al., 2012a,b; Dolk et al., 2012, 2013, 2014).

## Conclusion

Taken together, the present study demonstrates that participants flexibly adjust their allocation of attention based on the in-/dependence of receiving performance-contingent reward when interacting with others and thus to the contextual specificity of social interactions. Rather than fostering the consideration and differentiation of the other person, as happens when the relationship is characterized by cooperative dependence, pushing one’s own performance to the limit appears to be the suitable strategy in a competitive context. Therefore, people keep their eyes on themselves when aiming at beating a co-actor and emerging as the winner.

## Author Contributions

All authors contributed equally to study design, data analysis, drafts and revisions of the manuscript. In addition, JM was responsible for data collection.

## Conflict of Interest Statement

The authors declare that the research was conducted in the absence of any commercial or financial relationships that could be construed as a potential conflict of interest.

## Footnotes

1 In order to select the sample size, we entered Experiment 2 of the study by Iani et al. (2011) into the following website https://designingexperiments.shinyapps.io/BUCSS_ss_power_spa/. Considering the design (i.e., two-factor mixed ANOVA), sample-size (i.e., *N* = 32), observed *F*-value (i.e., *F* = 8.82), number of levels for between-subjects (i.e., two) and within-subjects factors (i.e., two), the effect of interest (i.e., the interaction between both factors), the alpha-level for the previous and current study (i.e., both 0.05), a desired level of assurance of 0.5 (i.e., correcting for publication bias) as well as a desired level of statistical power of 0.8 revealed a sample size of 21 participants per group. However, based on the findings by Liepelt et al. (2011) for sequential trial-by-trial adjustments using 24, we decided to also test 24 participants per group.

2 The difference between the number of male and female participants could bias the internal validity of the present study. Given, however, that most studies investigating the influence of interdependency on go–nogo Simon task performance had comparable female samples (i.e., Ruissen and de Bruijn, 2016: 92%; Hommel et al., 2009: 89%) and the fact that the manipulation of interdependency can be considered to be more effective in males as compared to females (Van Vugt et al., 2007), the impact of the present unbalanced gender-sample should (if any) further strengthen the present results in a balanced gender-sample.

3 Used studies (in alphabetic order): Colzato et al. (2012a): Independent group, interdependent group; Colzato et al. (2013): Convergent group, divergent group; Dittrich et al. (2012) and Costantini et al. (2013): Experiment 2 (horizontal joint go–nogo condition), Experiment 3 (horizontal joint go–nogo condition); Dittrich et al. (2017): Partition group, no partition group; Ferraro et al. (2011): Experiment 1; Hommel et al. (2009): Positive confederate, negative confederate; Iani et al. (2011): Experiment 1 (same group, different group), Experiment 2 (positive interdependence, negative interdependence); Iani et al. (2014): Experiment 1, Experiment 2; Klempova and Liepelt (2017): Experiment 1 (joint go–nogo condition), experiment 2 (joint go–nogo condition); Lam and Chua (2010): Joint go–nogo different alternative condition; Liepelt et al. (2011), Malone et al. (2014), and Ruissen and de Bruijn (2016): Solo condition, competitive condition, cooperative condition; Sebanz et al. (2003): Experiment 1, Experiment 2 (no feedback group); Stenzel et al. (2014): Experiment 1 (agency+/intentionality+ condition); Yamaguchi et al. (2016).
