# Supplementary material for: Are You Keeping an Eye on Me? The Influence of Competition and Cooperation on Joint Simon Task Performance
Source: Front Psychol. 2018 Aug 3;9:1361. doi: 10.3389/fpsyg.2018.01361 (PMC6085593; doi:10.3389/fpsyg.2018.01361)
Supplement: Supplementary file 2 [file Table_2.DOCX]

Supplementary Material

Are you keeping an eye on me? The influence of competition and cooperation on joint Simon task performance

Jonathan Mendl, Kerstin Fröber, Thomas Dolk

Department of Experimental Psychology, University of Regensburg, Germany

*** Correspondence:** Thomas Dolk, Department of Experimental Psychology, University of Regensburg, Universitätsstraße 31, 93053 Regensburg, Germany. E-mail: thomas.dolk@ur.de.

# Table S2: ANOVA results comparing Task 2 and 3: 2(Group) x 2(Task 2 vs. Task 3) x 2(Block) x 2(Transition) x 2(Compatibility) x 2(CompatibilityN-1)

| Effect/Interaction | *F*(1, 43) | *p* value | η_p_² |
| --- | --- | --- | --- |
| Group | 1.84 | .182 | .04 |
| Task | 1.77 | .190 | .04 |
| Task x Group | < 0.01 | .958 | < .01 |
| Block | 52.82 | < .001 | .55 |
| Block x Group | 1.33 | .256 | .03 |
| **Transition** | **52.82** | **< .001** | **.55** |
| Transition x Group | 1.29 | .263 | .03 |
| **Compatibility** | **9.99** | **< .01** | **.19** |
| Compatibility x Group | 0.04 | .845 | < .01 |
| CompatibiltiyN-1 | 0.71 | .404 | .02 |
| CompatibilityN-1 x Group | 0.39 | .538 | .01 |
| Task x Block | 0.57 | .454 | .01 |
| Task x Block x Group | < 0.01 | .995 | < .01 |
| **Task x Transition** | **4.92** | **< .05** | **.10** |
| Task x Transition x Group | 0.10 | .752 | < .01 |
| **Block x Transition** | **10.74** | **< .01** | **.20** |
| Block x Transition x Group | 0.15 | .702 | < .01 |
| Task x Block x Transition | 3.12 | .084 | .07 |
| Task x Block x Transition x Group | < 0.01 | .982 | < .01 |
| Task x Compatibility | 0.07 | .798 | < .01 |
| Task x Compatibility x Group | 1.82 | .184 | .04 |
| Block x Compatibility | 0.73 | .398 | .02 |
| Block x Compatibility x Group | 0.82 | .370 | .02 |
| Task x Bock x Compatibility | 0.01 | .906 | < .01 |
| Task x Block x Compatibility x Group | 0.40 | .531 | .01 |
| Transition x Compatibility | 0.03 | .861 | < .01 |
| Transition x Compatibility x Group | 0.30 | .589 | .01 |
| Task x Transition x Compatibility | 2.03 | .161 | .05 |
| Task x Transition x Compatibility x Group | 1.36 | .250 | .03 |
| Block x Transition x Compatibility | < 0.01 | .983 | < .01 |
| Block x Transition x Compatibility x Group | 0.37 | .55 | .01 |
| **Task x Block x Transition x Compatibility** | **5.30** | **< .05** | **.11** |
| Task x Block x Transition x Compatibility x Group | 0.01 | .941 | < .01 |
| Task x CompatibilityN-1 | 3.72 | .060 | .08 |
| Task x CompatibilityN-1 x Group | 0.32 | .574 | .01 |
| Block x CompatiblityN-1 | 3.42 | .071 | .074 |
| Block x CompatibilityN-1 x Group | 0.29 | .594 | .01 |
| Task x Block x CompatibilityN-1 | 0.40 | .530 | .01 |
| **Task x Block x CompatibilityN-1 x Group** | **4.67** | **< .05** | **.09** |
| **Transition x CompatibilityN-1** | **12.17** | **< .01** | **.22** |
| Transition x CompatibilityN-1 x Group | 0.08 | .784 | < .01 |
| Task x Transition x CompatibilityN-1 | 0.46 | .501 | .01 |
| Task x Transition x CompatibilityN-1 x Group | < 0.01 | .983 | < .01 |
| Block x Transition x CompatibilityN-1 | 1.52 | .224 | .03 |
| Block x Transition x CompatibilityN-1 x Group | 1.36 | .250 | .03 |
| Task x Block x Transition x CompatibilityN-1 | < 0.01 | .990 | <.01 |
| Task x Block x Transition x CompatibilityN-1 x Group | 2.61 | .113 | .06 |
| **Compatibility x CompatibilityN-1** | **272.66** | **< .001** | **.86** |
| Compatibility x CompatibilityN-1 x Group | 1.40 | .243 | .03 |
| Task x Compatibility x CompatibilityN-1 | 2.03 | .161 | .05 |
| Task x Compatibility x CompatibilityN-1 x Group | .23 | .635 | .01 |
| Block x Compatibility x CompatibilityN-1 | .311 | .580 | .01 |
| **Block x Compatibility x CompatibilityN-1 x Group** | **4.61** | **< .05** | **.10** |
| Task x Block x Compatibility x CompatibilityN-1 | 0.01 | .931 | < .01 |
| Task x Block x Compatibility x CompatibilityN-1 x Group | < 0.01 | .956 | < .01 |
| **Transition x Compatibility x CompatibilityN-1** | **78.10** | **< .001** | **.65** |
| Transition x Compatibility x CompatibilityN-1 x Group | 1.62 | .210 | .04 |
| Task x Transition x Compatibility x CompatibilityN-1 | 0.06 | .810 | < .01 |
| Task x Transition x Compatibility x CompatibilityN-1 x Group | 1.74 | .195 | .04 |
| Block x Transition x Compatibility x CompatibilityN-1 | 0.36 | .553 | .01 |
| Block x Transition x Compatibility x CompatibilityN-1 x Group | < 0.01 | .993 | < .01 |
| **Task x Block x Transition x Compatibility x CompatibilityN-1** | **4.14** | **< .05** | **.09** |
| Task x Block x Transition x Compatibility x CompatibilityN-1 x Group | 0.42 | .523 | .01 |

Note: Bold indicates a significant effect at alpha = .05
